# Prevalence and associated factors of nosocomial infection among children admitted at Jimma Medical Center, Southwest Ethiopia: a retrospective study

**DOI:** 10.3389/fped.2025.1485334

**Published:** 2025-04-04

**Authors:** Serkalem Aschalew Jima, Tamirat Beyene Gerete, Fikadu Balcha Hailu, Girma Bacha Ayane, Merga Garoma Jatu, Temesgen Geta Hardido, Elias kenea Tolosa

**Affiliations:** ^1^ Department of Nursing, College of Health Sciences, Arsi University, Asella, Ethiopia; ^2^ School of Midwifery, College of Health Sciences and Medicine, Wolaita Sodo University, Wolaita Sodo, Ethiopia; ^3^ School of Nursing, Institute of Health Sciences, Jimma University, Jimma, Ethiopia; ^4^ Department of Nursing, Institute of Health Sciences, Dambi Dollo University, Dambi Dollo, Ethiopia; ^5^ School of Nursing, College of Health Sciences and Medicine, Wolaita Sodo University, Wolaita Sodo, Ethiopia; ^6^ Department of Nursing, Hossana College of Health Sciences, Hossana, Ethiopia

**Keywords:** prevalence, nosocomial infection, children, Ethiopia, Jimma

## Abstract

**Background:**

Nosocomial infections are a significant public health problem worldwide, affecting hundreds of millions of patients annually. However, studies on nosocomial infections specifically focused on pediatric patients in Ethiopia are limited. Therefore, this study aimed to assess the prevalence of nosocomial infections and associated factors among children admitted at Jimma Medical Center, southwest Ethiopia.

**Methods:**

An institution-based retrospective cross-sectional study design was conducted from June 1 to 30, 2023. Data were collected from the medical records of children. A systematic random sampling technique was employed to select a total of 417 medical records. Data were collected using structured checklists. The collected data were entered into Epi-data version 4.6, and Statistical Package for Social Science version 26.0 was used for analysis. The variables with a *p*-value less than 0.05 were considered as statistically significant.

**Results:**

A total of 417 (92.87%) medical records of the pediatric patient the inclusion criteria. Of these, 99 (23.74%) of pediatric patients developed nosocomial infections. Malnutrition [AOR = 2.01; 95% CI: 1.18, 3.42], length of hospital stay [AOR = 3.19; 95% CI: 1.73, 5.90], antibiotics received at admission [AOR = 4.76; 95% CI: 1.86, 12.15], being on mechanical ventilation [AOR = 5.04; 95% CI: 2.44, 10.43], blood transfusion [AOR = 4.51; 95% CI: 2.43, 8.35], and urinary catheter [AOR = 3.26; 95% CI: 1.72, 6.18] were significantly associated.

**Conclusion:**

The findings of this study indicated that nearly a quarter of children developed nosocomial infections. Malnutrition, length of hospital stay, antibiotics received at admission, being on mechanical ventilation, urinary catheter, and blood transfusion contributed to the development of nosocomial infections. Therefore, the concerned bodies should immediately prevent nosocomial infections and improve identified factors.

## Introduction

Nosocomial infections, also known as hospital-acquired infections, are infections that patients acquire while receiving healthcare in hospitals or other healthcare facilities (1). These infections typically manifest after 48 h of admission and are not present or incubating at admission (2). Various microorganisms contribute to nosocomial infections, with bacteria responsible for approximately 90% of cases, while viruses, fungi, and protozoa are less common culprits (3). The most prevalent types of nosocomial infections include bloodstream infections, urinary tract infections, surgical site infections, and pneumonia (4, 5).

The global impact of nosocomial infections is profound, affecting hundreds of millions of patients annually (6) and imposing significant economic burdens on healthcare systems (7). Incidence rates range from 5.7% to 19.1% in low- and middle-income countries and from 3.5% to 12% in developed nations (8).

Pediatric patients are particularly vulnerable due to factors such as immature immune systems, prolonged hospital stays, and nutritional challenges (9–11). The risk is exacerbated in developing countries, where inadequate infection prevention practices and increasing use of antimicrobial agents and medical devices heighten exposure (12, 13).

In high-income countries, nosocomial infections affect approximately 4%–8% of pediatric patients (14, 15). However, in African countries, the incidence is rising alarmingly due to negligence in adhering to infection prevention guidelines and insufficient healthcare funding (16). A study in Sub-Saharan Africa reported an incidence density of 26.7 infections per 1,000 pediatric patients per day (17).

Alarmingly, around 4.9 million children die from infectious diseases each year, with nosocomial infections contributing significantly to these fatalities (18). In neonatal populations, these infections account for between 4% and 56% of deaths among hospitalized babies (6). In Sub-Saharan Africa, 1.2 million newborns die before reaching 28 days of age, with a substantial portion of these deaths attributed to nosocomial infections (19).

The growing prevalence of nosocomial infections among pediatric patients raises concerns about increasing antibiotic resistance, prolonged hospital stays, and the financial burden on families and healthcare systems (18, 20–22). These infections are the most common complications faced by hospitalized children, often leading to emotional distress, functional disorders, and reduced quality of life (23).

While identifying the factors associated with nosocomial infections in pediatric patients is critical, there is a notable lack of studies focusing specifically on this demographic in Ethiopia, particularly at Jimma Medical Center. Key variables, such as immunization status and the variety of antibiotics used, have not been adequately investigated as predictors of nosocomial infections among pediatric patients. Therefore, this study aimed to assess the prevalence of nosocomial infections and their associated factors among children admitted to Jimma Medical Center in Southwest Ethiopia.

## Methods and materials

### Study setting and period

This research was carried out at Jimma Medical Center. Jimma Medical Center is the only tertiary hospital in the southwestern part of Ethiopia, located in Jimma town. 352 km from Addis Ababa, the capital city of Ethiopia. Being a well-established referral hospital, it provides for the diverse health needs of an estimated 20 million people living in that area. It provides services for approximately 15,000 inpatients and 160,000 outpatients. It can also serve 11,000 emergency cases and 4,500 deliveries per year. It has 800 beds and 1,600 staff members (24).

The Department of Pediatric and Child Health is one of the main units and contains 77 staff nurses, 1 pediatric oncologist, 2 senior surgeons, and 7 pediatricians. An average annual admission capacity of 3,564 patients, including the pediatric ward, pediatric oncology ward, PICU, and NICU. It contains 157 beds, including those in the pediatric intensive care unit, pediatric oncology ward, neonatal intensive care unit, and pediatric ward.

The study was conducted from June 1 to 30, 2023.

### Study design

An institutional-based retrospective cross-sectional study design was employed.

### Source population

All medical records of children who were admitted to Jimma Medical Center.

### Study population

All selected medical records of children who were admitted to Jimma Medical Center from January 1, 2020, to December 31, 2022.

### Inclusion criteria

All medical records of children who were admitted to Jimma Medical Center and stayed for more than 48 h were included.

### Exclusion criteria

Records of children with incomplete medical records and those that have been reported.

### Sample size determination

A single population proportion formula, $n=\frac{\left(\frac{z\alpha }{2}\right)p(1-p)}{d^{2}}$, was used to calculate the sample size for a specific objective. The margin of error (d) = 5%, 95% confidence level, and the proportion of nosocomial infection (p) = 21.4%, taken from a previous study (25). $n=\frac{\left({\left(1.96\right)}^{2}\;0.214(1-0.214)\right)}{0.05^{2}}=258.4$ By considering 10% of lost medical records of paediatric patients, the first objective sample size was 284.

To determine the required sample size for the second specific objective of this study, taking into account various factors that are significantly associated with the outcome variables with a confidence level of 95%, a margin of error of 5%, a power of 80%, and a ratio of 1:1, and by using the Open Epi Info 7.2.5 StatCalc software program for the double population proportions formula. The sample size calculated for the second objective was greater than that of the first objective (Table 1).

**Table 1:** Sample size determination using associated factors of nosocomial infection among children at Jimma Medical Center, Southwest Ethiopia, 2023.

Variable	Confidence level (%)	Power (%)	% Outcome in unexposed	AOR	Sample size (*n*)	After adding 10% of lost medical records	References
Length of hospital stay	95	80	12.3	4.1	114	125	(26)
Age	95	80	8.4	3.3	214	235	(26)
Nasogastric tube feeding	95	80	12.5	2.45	298	323	(26)
Duration Mechanical ventilation	95	80	10	3.46	172	189	(26)
Malnutrition	95	80	17.3	2.10	354	389	(25)
Use of urine catheter	95	80	64.4	1.9	408	449	(27)
Comorbidity	95	80	10.1	2.74	270	297	(28)

Therefore the largest sample size was taken to have maximum representativeness, so the final sample size was 449.

### Sampling procedure

To obtain the sample, medical record numbers were used as a sampling frame, sourced from the register logbook of pediatric wards, including the Pediatric Ward, Pediatric Intensive Care Unit (PICU), Neonatal Intensive Care Unit (NICU), and Pediatric Oncology Ward. A systematic random sampling technique was employed to select medical record numbers until the required sample size was achieved.

From January 1, 2020, to December 31, 2022, a total of 10,239 pediatric patients were admitted. The target sample size was determined to be 449. The sampling interval (K) was calculated by dividing the total admissions (10,239) by the target sample size (449), resulting in an interval of approximately 23. A random starting point between 1 and 23 was selected; for example, if the number 10 was chosen, the sample selection process began at this point. Subsequent medical record numbers were selected by advancing every 23rd unit until the required sample size was reached. The identified medical records were then retrieved from the medical record store for analysis.

### Study variables

Nosocomial infection was the outcome variable of this study. The socio-demographic characteristics (age and sex), clinical condition-related factors [malnutrition, comorbid condition, length of hospital stay, antibiotics received at admission, number of types of antibiotics received, place of admission (ward type), serostatus for HIV, anemia, immunization status, previous hospitalization], invasive medical devices, and other procedure-related factors [being on mechanical ventilation, duration of mechanical ventilation, urinary catheters, blood transfusions, surgical procedures, chest tubes, IV cannulas, and nasogastric tubes (NGT)] were independent variables.

### Operational definition

#### Nosocomial infection

An infection acquired by a patient during their stay in a hospital or other healthcare facility. This infection must not have been present or incubating at the time of admission and should occur after 48 h of admission (29, 30).

#### Incomplete medical record

Refers to medical records that lack essential information, such as date of admission or discharge, or do not contain at least one independent variable.

#### Malnutrition

An imbalance between the nutrients an individual's body needs and the nutrients they consume. This imbalance can be indicated by a weight-for-height ratio of fewer than −2 *z*-scores or a Middle Upper Arm Circumference (MUAC) measurement of less than 12.5 cm.

#### Invasive medical devices

Any medical device that is introduced into the body through a break in the skin or another entry point, allowing it to penetrate the body.

## Data collection tools and techniques

The data collection tools were adapted after a review of different relevant literature (25–27, 29, 31–34). The tools were prepared in the English language. The checklist consists of three parts: sociodemographic characteristics, clinical condition-related factors, and invasive medical device procedure-related factors Supplementary File 1.

The data collection process was carried out by three Bachelor of Science nurses with prior experience in data collection who reviewed the medical records meeting the inclusion criteria and extracted the necessary information. The data collection process was supervised by a principal investigator.

## Data quality assurance

Before data collection, training was given to data collectors and supervisors for one day on the purpose of data collection, the content of the checklist, where the medical records are located, and how to extract the necessary data from medical records. A pretest was conducted at Jimma Medical Center (from 2019 admission) by taking 5% (23) of the total sample size one week before the actual data collection. Based on the pretest results, necessary modifications were made, such as removing items that could not be found in the medical records. The supervisor monitored the data collection process daily. Additionally, the principal investigator and supervisor reviewed the complete checklists filled out by the data collectors to ensure the completeness and consistency of the collected information.

## Data processing and analysis

Before analysis, the data were cleaned, verified, and coded. The cleaned data were then imported into Epidata version 4.6 and exported to SPSS version 26 for analysis. Descriptive statistics were initially computed to summarize the data characteristics. To assess linear correlations between independent variables, inferential statistics were employed. Multicollinearity was evaluated using tolerance and variance inflation factors (VIF). Variables exhibiting a VIF greater than 10 or a tolerance less than 0.1 were excluded from further analysis. Specifically, the VIF values ranged from less than 2.825, and tolerance values were above 0.354. The study utilized binary logistic regression to explore the relationship between dependent and independent variables. Additionally, multivariable logistic regression analysis was conducted to identify factors associated with nosocomial infections in pediatric patients. The model's fit was assessed through the omnibus test (*p* = 0.00), and Hosmer and Lemeshow's test indicated a good fit (*p* = 0.770). To gauge the strength of correlations, odds ratios and 95% confidence intervals were calculated. The statistical significance threshold was set at a *p*-value of less than 0.05. The findings were presented using appropriate tables, charts, figures, graphs, and narrative prose to ensure clarity and comprehensibility.

## Ethical approval

This research was conducted in keeping with the Helsinki Declaration. Ethical approval for this study was obtained from the Ethical Review Committee of Jimma University (Ref No: JUIH 414/23). Subsequently, a letter was submitted to the administration of Jimma Medical Center (JMC) to obtain permission before the actual data collection. The information gathered from medical records was confidential and used only for research purposes.

## Results

### Socio-demographic characteristics of children

Out of the reviewed 449 medical records, 417 met the inclusion criteria, with a response rate of 92.87%. Of the admitted pediatric patients, 148 (35.5%) were aged ≤28 days. 257 (61.6%) pediatric patients were male (Table 2).

**Table 2:** Socio-demographic characteristics of children admitted at Jimma Medical Center, Southwest Ethiopia, 2023.

Variables	Category	Frequency (%)
Age	≤28 days	148 (35.5)
	29 days–11 months	57 (13.7)
	1–3 years	77 (18.5)
	4–6 years	68 (16.3)
	7–12 years	43 (10.2)
	≥13 years	24 (5.8)
Sex	Male	257 (61.6)
	Female	160 (38.4)

### Clinical characteristics of children

Out of a total of 417 samples, 236 (56.6%) were admitted to the pediatric ward. About 139 (33.3%) of pediatric patients had a history of previous hospitalizations. 388 (93.0%) were received antibiotics at admission. Of this, 339 (81.3%) had received less than or equal to three types of antibiotics. 124 (29.7%) were malnourished, 67 (16.1%) were diagnosed with anemia, 20 (4.8%) were HIV positive, and 88 (21.1%) had comorbid diseases. 260 (62.4%) of pediatric patients stayed in the hospital for more than seven days (Table 3).

**Table 3:** Clinical characteristics of children admitted to Jimma Medical Center, Southwest Ethiopia, 2023.

Variables	Category	Frequency (%)
Place of admission (ward type)	NICU	148 (35.5)
	PICU	16 (3.8)
	Pediatric oncology ward	17 (4.1)
	Pediatric ward	236 (56.6)
Previous hospitalization	Yes	139 (33.3)
	No	278 (66.7)
Antibiotic received at admission	Yes	388 (93.0)
	No	29 (7.0)
Number of types of antibiotic received at admission	≤3	339 (81.3)
	>3	49 (11.7)
Malnutrition	Yes	124 (29.7)
	No	293 (70.3)
Anaemia	Yes	59 (14.1)
	No	358 (85.9)
Serostatus for HIV	Reactive	20 (4.8)
	Non-reactive	397 (95.2)
Comorbidity	Yes	88 (21.1)
	No	329 (78.9)
Length of hospital stay	>7 days	260 (62.4)
	≤7days	157 (37.6)

### Immunization status of children

The findings revealed that 148 (35.5%) were fully vaccinated. 201 (48.20%) were vaccinated according to their age. About 13 (3.10%) were partially vaccinated and 55 (13.20%) not vaccinated (Table 4).

**Table 4:** Immunization status of children admitted to Jimma Medical Center, Southwest Ethiopia, 2023.

Variable	Category	Frequency (%)
Immunization status	Fully vaccinated	148 (35.5)
	Vaccinated for age	201 (48.20)
	Parcially vaccinated	13 (3.10)
	Not vaccinated	55 (13.20)

### Invasive medical devices and procedure characteristics of children

In a study of pediatric patients, 405 (97.1%) were found to have used invasive medical devices. Among these, 391 (93.8%) had a history of IV cannulas, 19 (4.6%) had chest tubes, and 279 (66.9%) had nasogastric tubes. Additionally, 45 (10.8%) of the pediatric patients had been on mechanical ventilation. Of these, 22 (5.3%) were on mechanical ventilation for seven days or more. Furthermore, 58 (13.9%) had a history of urinary catheters, and 106 (25.4%) underwent surgical procedures after admission. 67 (16.1%) had a history of blood transfusion (Table 5).

**Table 5:** Invasive medical device and other procedure procedures for children admitted to Jimma Medical Center, Southwest Ethiopia, 2023.

Variables	Category	Frequency (%)
Presence of invasive medical devices and procedure	Yes	405 (97.1)
	No	12 (2.9)
IV Cannula	Yes	391 (93.8)
	No	26 (6.2)
Chest tube	Yes	19 (4.6)
	No	398 (95.4)
Nasogastric tubes (NGT)	Yes	279 (66.9)
	No	138 (33.1)
Mechanical ventilation	Yes	45 (10.8)
	No	372 (89.2)
Duration of mechanical Ventilation	<72 h	14 (3.3)
	4–6 days	9 (2.2)
	≥7 days	22 (5.3)
Urinary catheters	Yes	58 (13.9)
	No	359 (86.1)
Surgical procedures	Yes	106 (25.4)
	No	311 (74.6)
Blood transfusion	Yes	67 (16.1)
	No	350 (83.9)

### Laboratory findings and bacteriological profiles of children

Cultures were performed for 97 (23.3%) pediatric patients who developed nosocomial infections, of which 91 (21.8%) had isolated organisms. The most commonly isolated organisms were Klebsiella pneumonia, 30 (7.2%), followed by Staphylococcus aureus, 22 (5.3%) (Table 6).

**Table 6:** Laboratory finding and bacteriological profile of children admitted to Jimma Medical Center, Southwest Ethiopia, 2023.

Variables	Category	Frequency (%)
Was culture done?	Yes	97 (23.3)
	No	320 (76.7)
If yes, was organism grow from the culture?	Yes	91 (21.8)
	No	6 (1.5)
Isolated organism	CONS	21 (5)
	Klebsilla Pneuminae	30 (7.2)
	Enterococcus	7 (1.7)
	Staphylococcus aureus	22 (5.3)
	Klebsiella oxytoca	8 (1.9)
	Others*, specify	3 (0.7)

* Others:- Escherichia coli.

### Prevalence of nosocomial infection among children

**Figure 1:**
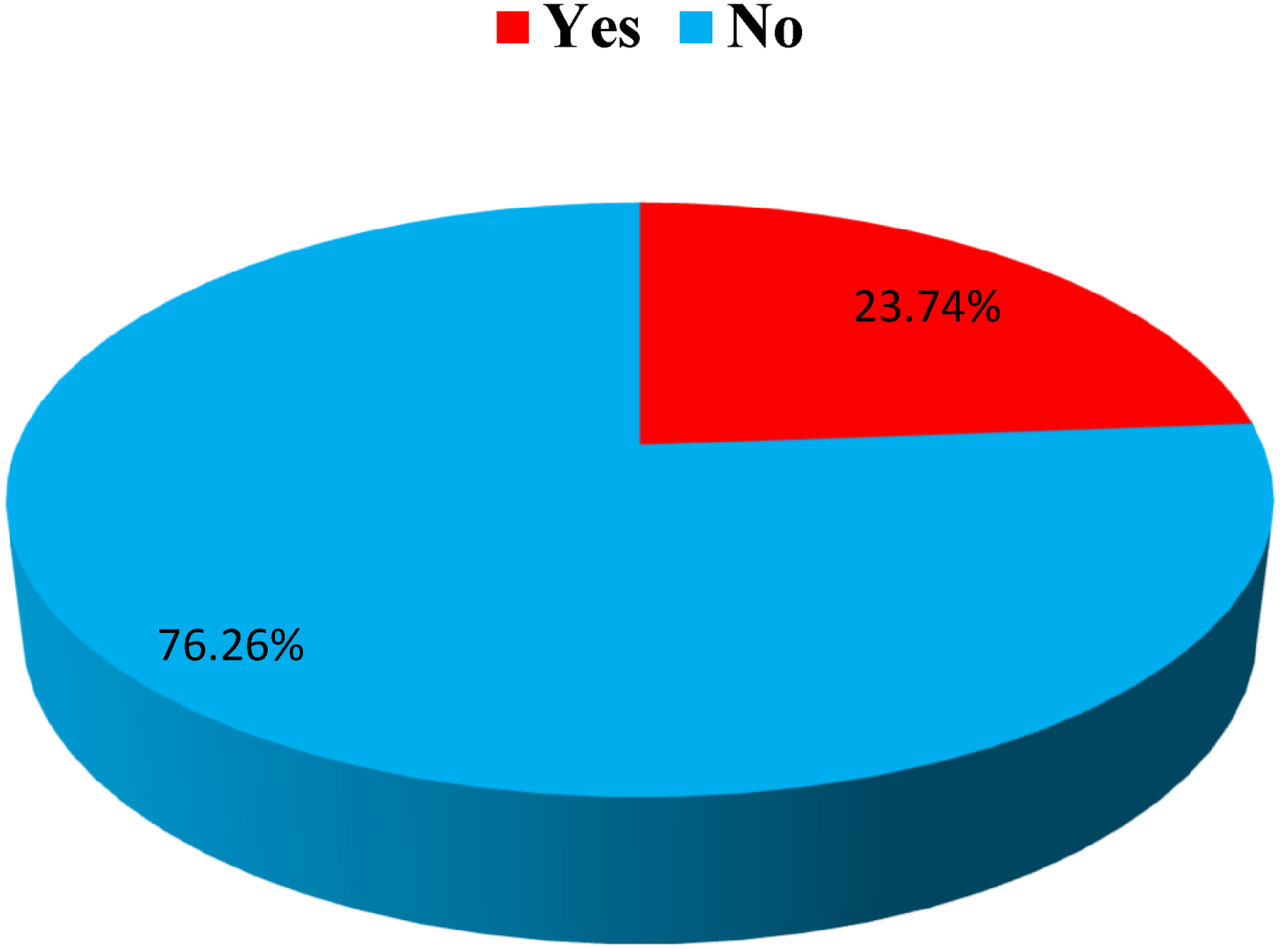
Prevalence of Nosocomial Infections Among children at Jimma Medical Center, 2023.

The overall prevalence of nosocomial infections among children in the current study was 23.74% (95% CI; 19.64, 27.84) (Figure 1).

### Types of nosocomial infections among children

**Figure 2:**
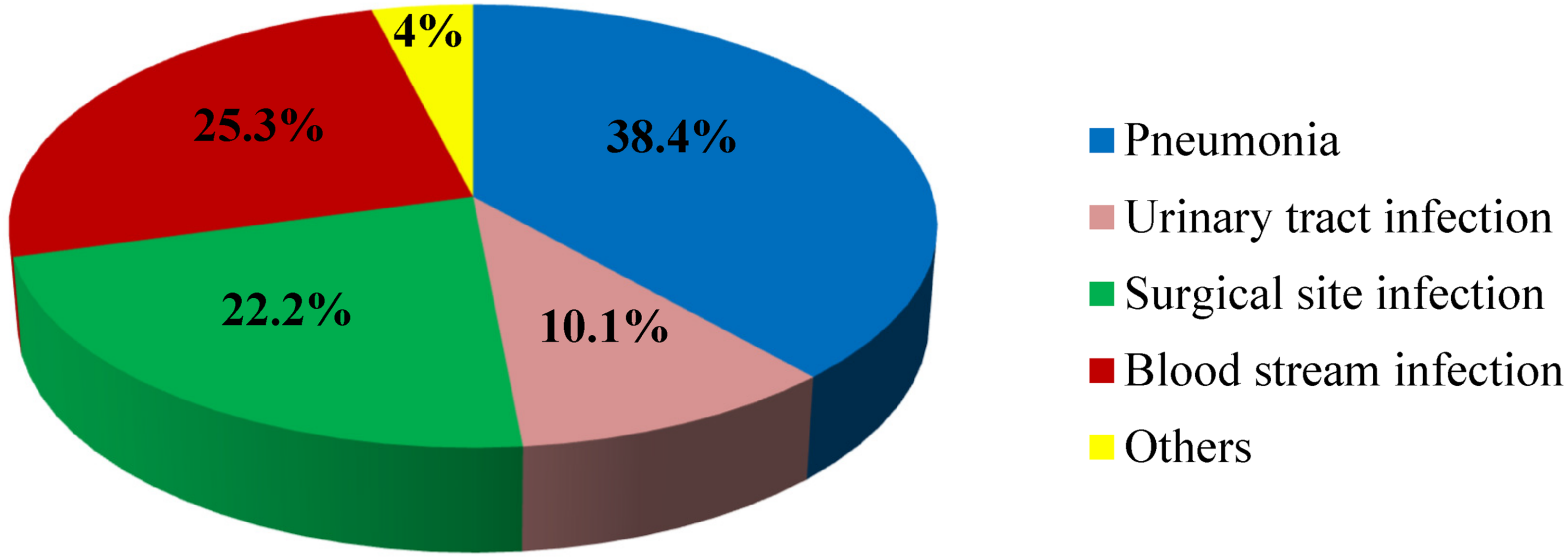
Types of nosocomial infections Among children at Jimma Medical Center, 2023. *Others: skin infection, meningitis, and gastrointestinal infection.

The most common type of nosocomial infection among children observed in the current study was pneumonia, which accounts for about 38.4% (Figure 2).

#### Factors associated with nosocomial infection among children

On multivariable logistic regression analysis, six variables were found to be statistically significant predictors of nosocomial infection among children, with a *p*-value of <0.05 and an AOR of 95% CI. Accordingly, malnutrition, length of hospital stay, antibiotic received at admission, being on mechanical ventilation, having a blood transfusion, and having a history of urinary catheters were the final independent predictors of nosocomial infections among children.

The odds of developing nosocomial infections among children who were malnourished were 2.01 times higher compared to those who were well-nourished [AOR = 2.01; 95% CI (1.18, 3.42); *P* = 0.010]. The odds of developing nosocomial infections among children who stay in the hospital for more than seven days were 3.19 times higher compared to those with a stay of seven days or less [AOR = 3.19; 95% CI (1.73, 5.90); *P* < 0.001]. The odds of developing nosocomial infections among children who received antibiotics at admission were 4.76 times higher compared to those who did not receive antibiotics at admission [AOR = 4.76; 95% CI (1.86, 12.15); *P* = 0.001].

The odds of developing nosocomial infections among children who had mechanical ventilation were 5.04 times higher compared to those without mechanical ventilation [AOR = 5.04; 95% CI (2.44, 10.43); *P* < 0.001]. The odds of developing nosocomial infections among children who received blood transfusions were 4.51 times higher compared to children who did not receive blood transfusions [AOR = 4.51; 95% CI (2.43, 8.35); *P* < 0.001]. The odds of developing nosocomial infections among children who had urinary catheters were 3.26 times higher compared to non-urinary catheterized children [AOR = 3.26; 95% CI (1.72, 6.18); *P* = 0.014] (Table 7).

**Table 7:** The multivariable logistic regression factors associated with nosocomial infection among children admitted to Jimma Medical Center, Southwest Ethiopia, 2023.

Variable	Category	Nosocomial infection		COR (95% CI)	*P***-**value	AOR (95% CI)	*P***-**value
		No (%)	Yes (%)				
Age	≤28 days	121 (81.8)	27 (18.2)	3.20 (1.28, 7.97)	0.004	1.82 (0.63, 5.23)	0.170
	29 days–11 Months	34 (59.9)	23 (40.4)	1.06 (0.40, 1.28)	0.012	0.85 (0.28, 2.58)	0.077
	1–3 years	64 (83.1)	13 (16.9)	3.52 (1.28, 9.63)	0.912	2.38 (0.76, 7.47)	0.536
	4–6 years	54 (79.4)	14 (20.6)	2.75 (1.01, 7.50)	0.014	2.06 (0.65, 6.51)	0.785
	7–12 years	31 (72.1)	12 (27.9)	1.85 (0.65, 5.27)	0.047	1.07 (0.32, 3.60)	0.275
	≥13 years	14 (58.3)	10 (41.7)	1	0.253	1	0.268
Comorbidity	Yes	258 (78.4)	71 (21.6)	1.69 (1.01, 2.85)	0.046	1.33 (0.72, 2.44)	0.363
	No	60 (68.2)	28 (31.8)	1		1	
Anaemia	Yes	40 (67.8)	19 (32.2)	0.61 (0.33, 1.10)	0.102	1.55 (0.73, 3.29)	0.257
	No	278 (77.7)	80 (22.3)	1		1	
Malnutrition	Yes	81 (65.3)	43 (34.7)	0.45 (0.28, 0.71)	0.001	**2.01** **(1.18, 3.42)**	**0**.**010**
	No	237 (80.9)	56 (19.1)	1		**1**	
Length of hospital stay	>7 days	180 (69.2)	80 (12.1)	0.31 (0.18, 0.54)	<0.001	**3.19** **(1.73, 5.90)**	**<0**.**001**
	≤7days	138 (87.9)	19 (30.8)	1		**1**	
Antibiotic received	Yes	300 (77.3)	88 (22.7)	2.08 (0.95, 4.57)	0.068	**4.76** **(1.86, 12.15)**	**0**.**001**
	No	18 (62.1)	11 (37.9)	1		**1**	
Mechanical ventilation	Yes	23 (51.1)	22 (48.9)	0.27 (0.14, 0.52)	<0.001	**5.04** **(2.44, 10.43)**	**<0**.**001**
	No	295 (79.3)	77 (20.7)	1		**1**	
Surgical procedure	Yes	73 (68.9)	33 (31.1)	0.59 (0.36, 0.97)	0.040	1.17 (0.61, 2.26)	0.642
	No	245 (78.8)	66 (21.2)	1		1	
Chest tubes	Yes	12 (63.2)	7 (36.8)	0.52 (0.19, 1.35)	0.176	1.14 (0.33, 3.93)	0.831
	No	306 (76.9)	92 (23.1)	1		1	
Blood transfusion	Yes	35 (52.2)	32 (47.8)	0.26 (0.15, 0.45)	<0.001	**4.51** **(2.43, 8.35)**	**<0**.**001**
	No	283 (80.9)	67 (19.1)	1		**1**	
Urinary catheter	Yes	30 (51.7)	28 (48.3)	0.26 (0.15, 0.47)	<0.001	**3.26** **(1.72, 6.18)**	**<0**.**001**
	No	288 (80.2)	71 (19.8)	1		**1**	
Place of admission (Ward type)	NICU	121 (81.8)	27 (18.2)	1.39 (0.83, 2.33)	0.005	1.33 (0.32, 5.57)	0.712
	PICU	9 (56.3)	7 (43.8)	0.40 (0.14, 1.12)	0.082	2.89 (0.08, 7.96)	0.696
	Paediatric oncology ward	8 (47.1)	9 (52.9)	0.28 (0.10, 0.75)	0.012	0.54 (0.16, 0.85)	0.325
	Paediatric ward	180 (76.3)	56 (23.7)	1	0.205	1	0.555

1, Reference group.

The bold values are significantly associated variables at *P*-value <0.05.

## Discussion

This study, conducted at Jimma Medical Center in southwest Ethiopia, found that 23.74% (95% CI: 19.64, 27.84) of pediatric patients acquired nosocomial infections. Significant factors associated with these infections included malnutrition, prolonged hospital stays, antibiotics received at admission, mechanical ventilation, blood transfusions, and urinary catheterization.

The prevalence of nosocomial infections among children in the current study is consistent with previous studies conducted at Hawassa Comprehensive Specialized Hospital (21.4%) (25) and Ayder Hospital (20.2%) (26). However, it is higher when compared to previous studies conducted in Ecuador (13.5%) (35), Switzerland (6.7%) (28), the Philippines (11.37%) (36), the South of Iran (14.57%) (37), and Kenya (2.62%) (38). These discrepancies may stem from differences in inclusion criteria and study designs. For instance, studies in Ecuador and South Iran included patients older than one month, while our study encompassed patients from birth to 17 years. The Swiss study limited participants to those hospitalized for over 24 h, whereas our criteria were over 48 h. Additionally, we included patients from the pediatric oncology ward, unlike the studies from the Philippines and Kenya, which focused solely on pediatric wards, PICU, and NICU.

Conversely, our prevalence is lower than Turkey's report of 68.4% (39). likely due to their exclusive focus on bloodstream infections and a smaller sample size.

Malnutrition was shown to increase the odds of nosocomial infections by 2.01 times, consistent with findings from Ecuador, Indonesia, and Hawassa (25, 27, 35). This vulnerability can be attributed to compromised immune systems in malnourished children, reducing their ability to combat pathogens (40).

Pediatric patients with hospital stays exceeding seven days had 3.19 times higher odds of infection, supporting similar findings in Indonesia (27). Extended hospitalizations often indicate severe illness, increasing exposure to potential infections (41).

Antibiotics received at admission raised infection odds by 4.76 times, similar to results from Ayder Hospital (26). This association may relate to antibiotic overuse, contributing to resistance and subsequent infections (42).

Mechanical ventilation was associated with 5.04 times higher odds of nosocomial infections, consistent with findings from Turkey (43). The irritation and inflammation caused by endotracheal tubes may facilitate airway colonization, increasing infection risks (44).

Additionally, pediatric patients who received blood transfusions had 4.51 times higher odds of developing nosocomial infections compared to those who did not. This finding is supported by a study in South Africa (33). Blood transfusions may temporarily suppress the immune system, compromising the patient's ability to combat infections (45). Aseptic technique during the vein puncture process may also play a role.

Finally, patients with urinary catheters had 3.26 times higher odds of nosocomial infections compared to those without catheters, supported by studies in Switzerland and Indonesia (27, 28). This association may be due to the prolonged duration of catheterization and potential lapses in aseptic techniques (46).

## Strengths and limitations

The study included a new variable, immunization status, and the number of types of antibiotics, which had not been previously investigated in Ethiopia. However, since the study relied on secondary data, some crucial independent variables were missing. Furthermore, the findings are limited in their generalizability since the study was conducted solely at Jimma Medical Center.

## Conclusion and recommendation

The findings of this study revealed that nearly a quarter of children developed nosocomial infections. Factors such as malnutrition, length of hospital stay, antibiotics received at admission, being on mechanical ventilation, having a blood transfusion, and a history of urinary catheterization were identified as significant predictors of nosocomial infections among children. Hence, the concerned bodies should focus intervention strategies on contributing factors to nosocomial infections among children. Upcoming researchers should conduct a prospective cohort study to incorporate variables not addressed in a retrospective study.

## Data Availability

The original contributions presented in the study are included in the article/Supplementary Material, further inquiries can be directed to the corresponding author.
