# Supplementary material for: Prevalence and associated factors of nosocomial infection among children admitted at Jimma Medical Center, Southwest Ethiopia: a retrospective study
Source: Front Pediatr. 2025 Apr 4;13:1485334. doi: 10.3389/fped.2025.1485334 (PMC12006196; doi:10.3389/fped.2025.1485334)
Supplement: Supplementary file 1 [file Supplementaryfile1.pdf]

Checklist

The checklist to assess the prevalence of nosocomial infection and associated factors among children in Jimma Medical Center, Southwest Ethiopia, 2023.

Instruction: Circle the answers, write answers to specify, and fill in the blank space.

**PART.1: Socio-demographic factors**			
S.No	Items	Response	Remark
101	Date of admission	_____/______/_____ (EC/EC)	
102	Age on date of admission	_______________day/month/year	
103	Sex	1. Male<br>2. Female	

**PART 2: Clinical condition-related factors**			
S.No	Items	Response	Remark
201	Does the child had a co-morbid disease at the time of admission?	1. Yes<br>2. No	
202	Does the child had anaemia?	1. Yes<br>2. No	
203	What was Serostatus for HIV of the child?	1. Reactive<br>2. Non-reactive	
204	Was the child malnourished?	1. Yes<br>2. No	
205	What was the immunization Status of the child?	1. Fully vaccinated<br>2. Vaccinated for age<br>3. Partially vaccinated<br>4. Not vaccinated	
206	Does the child have been Previous	1. Yes<br>2. No	

	hospitalization?		
207	Total length of hospital stay	1. >7 days<br>2. ≤ 7days	
208	Does the child received antibiotics at the time of admission?	1. Yes<br>2. No	If No Q.# 208 skip to Q.# 210
209	If yes Q.#208 Number of types of antibiotics received during admission	1. ≤ 3<br>2. >3	
210	Place of Admission(ward type)	1. NICU<br>2. PICU<br>3. Paediatric oncology ward<br>4. Paediatric ward	

**PART 3: Invasive medical devices and other procedure-related factors**			
S.No	Items	Response	Remark
301	Do any Invasive medical device procedures or other procedure was done for the child?	1. Yes<br>2. No	
If yes Q. # 301 what was the invasive medical device and procedure done for the child?			
302	Does the child have been on the mechanical ventilation?	1. Yes<br>2. No	If No Q.# 302 skip to Q.# 304
303	If yes for question No 302, how long has been on a Mechanical ventilation?	1. < 72 hours<br>2. 4–6 days<br>3. ≥7 days	
304	Does the child have history of Urinary catheters?	1. Yes<br>2. No	
305	After being admitted, was surgery performed?	1. Yes<br>2. No	
306	Does the child have been on a Chest tube?	1. Yes<br>2. No	

307	Does the child had an IV cannula?	1. Yes<br>2. No	
308	Have been a blood transfusion?	1. Yes<br>2. No	
309	Does the child have been on nasogastric tubes (NGT)?	1. Yes<br>2. No	

S.No	Items	Response	Remark
310	Does the child develop nosocomial infections (NIs)?	1. Yes<br>2. No	If No Q # 310 skip to Q # 312
311	If yes, Q .#310, what types of nosocomial infections?	1. Pneumonia<br>2. Urinary tract infection (UTI)<br>3. Surgical site infection (SSI)<br>4. Bloodstream infection(BSI)<br>5. Others, specify…..	
312	Was culcture done?	1. Yes<br>2. No	
313	If yes,was organism grow from the culture?	1. Yes<br>2. No	
314	If yes,Isolated organism	1. CONS<br>2. K.Pneuminae<br>3. Enterococcus<br>4. Staphylococcus aureus<br>5. Klebsiella oxytoca<br>6. Others,specify	
315	Date of discharge	_____/______/______(EC/GC)	
